# Functional status in relation to depression among elderly individuals in Indonesia: a cross-sectional analysis of the Indonesian National Health Survey 2018 among elderly individuals

**DOI:** 10.1186/s12889-021-12260-z

**Published:** 2021-12-30

**Authors:** Sri Idaiani, Lely Indrawati

**Affiliations:** National Institute of Health Research and Development, Ministry of Health of Republic of Indonesia, Jalan Percetakan Negara. 29, Jakarta, 10560 Indonesia

**Keywords:** Elderly, Depression, Functional status, 2018 basic health research

## Abstract

**Background:**

Depression among elderly individuals is related to physical illness, functional status, prolonged treatment and other factors. Depression is not effectively treated with medication but can be alleviated by treating the physical illness and improving functional status. Therefore, this study aims to determine the relationship between functional status and depression in elderly individuals in Indonesia.

**Methods:**

The data used were obtained from a national survey dataset, namely, the Basic Health Research and Socio-Economic Survey in 2018, which was carried out in 34 provinces and 514 districts or cities. The total number of respondents was 93,829, aged ≥60 years. Functional status and depression were assessed using the Barthel index and MINI (Mini International Neuropsychiatric Interview), respectively. Furthermore, data processing was carried out with the Statistical Package for Social Sciences (SPSS) version 26 program and analyzed using the chi-square test and multiple logistic regression with the complex sample method.

**Results:**

Elderly individuals with severe dependence were at the greatest risk of becoming depressed compared to those without functional impairment or with independence after considering sociodemographic factors and disease history have 5.730 (95% CI = 4.302-7.632) the adj odds. Furthermore, this figure was higher than that of individuals with total dependence have 4.147 (95% CI = 3.231-5.231) the adj odds. The physical illness experienced also played a role in the risk for depression, which was higher among elderly individuals with stroke and a history of injury. The sociodemographic factors involved include low education and economic levels. In contrast, the area of residence and marital status had no statistically significant effect on the relationship between functional disorders and depression.

**Conclusions:**

Elderly individuals with decreased functional status are prone to depression. The highest probability for depression was observed among those with severe dependence compared to those with total dependence. Consequently, interventions that involve various sectors, including social and family support, are needed.

**Supplementary Information:**

The online version contains supplementary material available at 10.1186/s12889-021-12260-z.

## Background

Indonesia is experiencing a transition towards an aging population due to the fact that the percentage of individuals over 60 years of age has reached over 7% of the total population. This condition makes the country appear to have an old population structure that has an increasing dependency ratio on the younger generation [1]. As an individual ages, health problems and diminished functional status arise, including cardiovascular diseases such as stroke, diabetes, joint disease, chronic lung disease and injuries [2]. In contrast to that in young individuals, depression in elderly individuals is associated with physical illness, functional status and prolonged care [3, 4].

Various studies have been carried out on the functional status and mental condition of elderly groups [5–8]. These studies assessed different conditions in certain populations, such as the elderly population in nursing homes or stroke survivors. No research has assessed the mental condition of elderly individuals in relation to their functional status in the general population [5–10]. Functional status is generally assessed through activities of daily living, and one of the mental conditions associated with this parameter is depression. This is because the mental condition of elderly individuals is closely related to low functional status [11]. Furthermore, deterioration in functional status puts individuals at risk for falls and other forms of injury [12, 13].

The prevalence of depression among elderly people varies, generally ranging from 7 to 30% [9, 10, 14]. However, it is higher in females, while for males, depression is strongly influenced by functional status, reduced mobility and ability to move at home [9, 10]. Furthermore, the prevalence of this condition is much higher when there are comorbid physical illnesses, especially chronic obstructive pulmonary disease, and drug treatment is generally ineffective and has low adherence [15].

In Indonesia, data regarding the functional status and depression of elderly individuals are available and were obtained from the 2018 National Health Survey (NHS) or in Indonesia, as this kind of work is more popular with basic health research (Riset Kesehatan Dasar, Riskesdas), which also contains data on chronic diseases. The annual NHSs that have been carried out for 5 years (2007, 2013) provided information on the health status of the population and risk factors for common diseases. Among the age groups of 55-64, 65-74 and ≥ 75 years, the prevalence of depression was 6.5, 8.5 and 8.9%, respectively, while for the age groups of 60-69, 70-79 and ≥ 80 years, the prevalence of total dependence was 1.07, 1.86 and 4.49%, respectively [16]. Although data on functional status and depression in elderly individuals are available, an in-depth assessment has yet to be carried out. Interesting to conduct an analysis of available data on the NHS because studies of depression and functional status in the general population are scarce. Therefore, this study aims to determine the effect of functional status on depression among elderly individuals in Indonesia by assessing characteristic variables and history of disease such as diabetes mellitus, heart disease, stroke, hypertension, rheumatism/joint disorders and injuries.

## Methods

### Design and data source

The data used were obtained from the NHS and National Socio-Economic Survey (Susenas) in 2018, which are national surveys routinely carried out by the Ministry of Health (National Institute of Health Research and Development) and Central Bureau of Statistics [16]. Furthermore, samples with national representation, even at the district/city level, were used. These two surveys were carried out in all provinces in Indonesia (34 provinces), with approximately 300,000 selected households spread across 514 districts/cities.

The NHS sampling frame was obtained in a multistage manner. The first and second stages determine census blocks, namely, selecting 30,000 census blocks from 25% of the master frame blocks of the most recent population census (in 2010). The selection was carried out by systematic probability proportional to size (PPS) random sampling in every urban and rural strata. The second stage selects 10 households from each CB that have been updated by systematic sampling with implicit stratification based on the highest education level of the head of the household to maintain representativeness among household characteristics. This approach is consistent with a previous article on the determinants of prediabetes in Indonesia, and the difference in the sampling frame for prediabetes is only in 26 provinces [17]. In the 30,000 census block, 10 households were identified, and all members of the selected households were asked to participate in this survey voluntarily [16].

The inclusion criteria included respondents ≥60 years of age, and answers personally provided not by a representative but by the closest individual in the house. Subjects were excluded if they are unable to communicate or unable to speak Indonesian. In principle, the subjects have to be able to communicate, the closest person can only help in terms of finding the right word equivalent. The elderly individuals fully represented by someone were still interviewed but in the questionnaire were coded as represent and would not be interviewed for depression questions.

### Instrument

Depression was assessed according to a section in the Mini International Neuropsychiatric Interview (MINI) version 6. This version refers to the International Classification of Diseases, tenth revision (ICD-10), which is still used in the disease coding system in Indonesia. Furthermore, the questionnaire used was developed in 1997 [18]. According to MINI version 6, subjects were categorized as depressed if they answered at least 2 “yes” from the first 3 questions plus 2 “yes” to questions 4 to 10. The MINI is a recognized instrument for assessing depression. Before being applied to the NHS 2018, an assessment of its validity and reliability was carried out, and good results were obtained [19]. Functional status was assessed by using the Barthel index, which is a measurement tool developed by Mahoney and Barthel [20] in 1965. This tool is widely used to assess the functional status of patients after stroke, colorectal cancer, fractures and several other medical and even social conditions in nursing homes and the general population [5, 6, 21–26]. A total of 10 questions were used to assess the respondents’ ability to perform their daily activities, including determining how much care and assistance was needed, and this assessment was carried out over a month. The criteria used included a score >/= 20 for the independent category, a score of 12-19 for the mild dependence category, a score of 9-12 for the moderate dependency category, a score of 5-8 for the severe dependence category and a score of 0-4 for the total dependence category. This stratification is based on the modified Barthel index [27]. Barthel index has been assessed for its validity and reliability in Indonesia [28].

Depression and functional status were assessed via face-to-face interviews by trained NHS enumerators. Interviews were conducted in the respondent’s house. Only elderly individuals residing at home were assessed; those living in nursing homes and those currently in hospitals were not included. The enumerators interviewed the respondents using a structured questionnaire in which there were questions, sociodemographic characteristics, history of chronic disease, depression and functional status. The Riskesdas report including questionnaire can be seen in Appendix 1.

Socioeconomic status was obtained from Susenas, which contains data describing household expenses. Susenas divides the socioeconomic status of Indonesian households into 5 groups. The expenditure quintile is the grouping of expenditures into five equal groups after being sorted from the smallest to the largest expenditure. The first quintile represents the poorest group, while the fifth quintile represents the richest group. Information about chronic diseases was obtained by via interviews. Participants were asked whether they suffer from hypertension, heart disease, or stroke; whether they have joints injuries confirmed by a doctor; and whether they suffered an injury that has interfered with daily activities in the last year.

### Data analysis

The age variable was grouped into 5-year intervals, while the other variables included sex (male or female), activity (not working or working/retired), marital status (married, divorced or unmarried), education (graduated from diploma1/diploma2/diploma3/higher education, no school, did not complete elementary school, graduated from junior high school or graduated from senior high school), residence (rural or urban) and economic status (upper, upper-middle, middle, lower-middle or low class). Furthermore, all disease histories were considered a nominal variable type (yes or no), which included diabetes mellitus (DM), heart disease, stroke, hypertension, joint disorders/rheumatism, and injury.

The prevalence of depression was calculated by considering the weighted value of the population and analyzed using the SPSS 24 (IBM SPSS Statistics) tool with a complex sample method. Furthermore, the relationship between the main independent variable, namely, functional disorders, was assessed by using the Barthel index, and the depression outcome variable was assessed by using the chi-square test. The Barthel index was an ordinal variable, while depression was a nominal variable (yes or no). In addition, the relationships of other independent variables to depression in the early stages were also assessed using the chi-square test. Moreover, the relationships between the depression variable and other independent variables were assessed using multiple logistic regression analysis. In the final stage, an assessment of the interactions and/or confounders between the main and independent variables was carried out.

A provincial map of Indonesia is displayed to show the provinces that have a high incidence of elderly individuals with severe dependency using the spmap command from STATA15 version (Stata Corp LLC).

### Ethical consideration

Ethical approval was obtained from the Research Ethics Commission of the NIHRD of Ministry of Health number LB 02.01/3/KE024/2018. All methods were carried out in accordance with the Helsinki Declaration. Subject participation was voluntary, and written informed consent was obtained from all participants prior to the interview.

## Results

### Participants

**Fig. 1:**
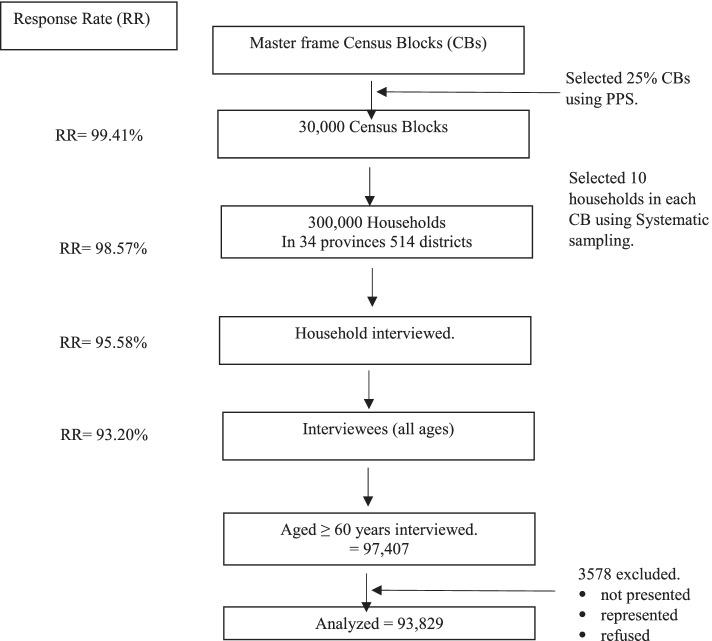
Flow chart of sampling stages

The response rate of CB visited, household visited, household interviewed was 99.41, 98.57 and 95.58%, respectively. The response rate of interviewees is 93.20%. The initial sample comprised 97,407 respondents, but after the selection process was carried out according to the study criteria, the sample size was 93,829. A total of 3578 respondents were not analysed because they were not present at the time of the survey or refused to be interviewed. See Fig. 1.

### Descriptive characteristics of respondents

Of the 93,829 respondents, 7491 or 7.7% (95% CI = 7.4-8.0) experienced depression, including 2829 males and 4662 females (two-thirds). As shown in Table 1, the marital status of the depressed respondents included over 4153 married, 3218 divorced (former spouses alive or dead) and 120 not married. The youngest respondent was 60 years old, and the average age was 68.48 years. Regarding residence area, the proportion of individuals residing in urban and rural areas was almost equal.

**Table 1:** Sociodemographic characteristics of the respondents

Characteristics	% Depression	95% CI	*p*-value
Age group (year)			
60-64	6.8	6.4 – 7.2	0.001
65-69	7.4	6.9 – 7.9	
70-74	9.0	8.2 – 9.7	
75-79	8.1	7.3 – 8.9	
80-84	9.5	8.4 – 10.8	
85-89	9.8	8.1 -11.8	
90+	12.0	9.1 – 15.6	
Sex			
Male	6.1	5.7 – 6.4	0.001
Female	9.2	8.8 – 9.6	
Occupation			
Unemployed	10.1	9.6 – 10.5	0.001
Employee/Retired	5.8	5.5 – 6.1	
Marital Status			
Married	6.8	6.5 – 7.2	0.001
Divorce/widowed	9.2	8.7 – 9.7	
Unmarried	8.2	6.1 – 11.6	
Education			
Diploma/Bachelor/University	3.5	2.7 – 4.4	0.001
Senior high school	5.0	4.4 – 5.8	
Junior high school	6.5	5.7 – 7.4	
Elementary school	8.0	7.5 – 8.5	
Did not finish elementary school	8.5	8.0 – 9.1	
No schooling	8.6	8.0 – 9.2	
Residence			
Rural	7.9	7.5 – 8.2	0.217
Urban	7.5	7.1 – 8.0	
Economic status			
Quintile 1	5.9	5.3 – 6.6	0.001
Quintile 2	6.9	6.3 – 7.7	
Quintile 3	7.8	7.1 – 8.6	
Quintile 4	7.5	6.9 – 8.2	
Quintile 5	8.8	8.3 – 9.4	

In terms of age range, the older the respondents were, the greater the proportion with depression. The highest proportion was within the age range of 90 years and above (12%). Regarding their activities, the proportion of individuals not working was greater than that of individuals working (Table 1). Moreover, the elderly individuals who were divorced, with their former spouses either alive or dead, comprised the largest proportion of depressed individuals compared to those still married to their spouse (husband/wife) or not married.

The proportion of elderly individuals who experience depression may increase with lower levels of education completed. It was discovered that depression is most common among those with no educational qualifications (8.6%). Regarding socioeconomic status, elderly individuals with a lower socioeconomic status comprised the largest proportion (8.8%) of depressed individuals compared to those with a higher socioeconomic status.

In the results section, the proportion of yes answers to the depression questions is also shown in Table 2.

**Table 2:** Percentage Depression items

	%	95% CI
1. Have you been consistently sad, depressed or down, most of the day, nearly every day?	14.26	14.24-14.69
2. Have you been most of the time less interested in most things or less able to enjoy the things you used to enjoy?	9.85	9.66-10.04
3. Did you feel tired or without energy, most of the time?	18.50	18.25-18.75
4. Did your appetite change significantly or did your weight increase or decrease without trying intentionally?	10.19	10.00-10.39
5. Did you have trouble sleeping nearly every night (difficulty falling asleep, night or early awakenings, hypersomnia)?	21.52	21.25-21.78
6. Did you talk or move more slowly than normal, or were you fidgety, restless or having trouble staying still?	9.80	9.61-9.99
7. Did you lose your self-confidence, or did you feel worthless or even inferior to other people?	4.59	4.46-4.73
8. Did you have feelings of self-reproach or guilt?	5.65	5.50-5.80
9. Did you have difficulty thinking or concentrating, or did you have trouble making decisions?	9.19	9.00-9.37
10. Did you consider hurting yourself, feel suicidal, or wish that you were dead?	0.97	0.91-1.04

The questions number 1 to 3 are filters therefore we focus on numbers 4 to 10. It shows the most symptoms that respondents experienced were sleep disturbances, disturbed appetite and limited movement.

Table 3 shows that the proportion of elderly individuals with an independent functional status comprised the smallest proportion of depressed (5.5%) individuals compared to those with functional disorders. In terms of functional disorders, elderly individuals with severe dependence represented the highest proportion of depression (36.3%). Furthermore, those experiencing chronic diseases such as diabetes mellitus, heart disease, stroke, hypertension, joint disorders/rheumatism and injuries comprised a greater proportion of patients with depression than those not experiencing such diseases.

**Table 3:** Association of depression with functional status & history of disease

	% Depression	95% CI	*p*-value
Functional Status:			
- Dependent	5.5	5.3 - 5.8	0.001
- Mild dependency	12.8	12.0 – 13.5	
- Moderate dependency	27.2	23.4 – 31.4	
- Severe dependency	36.3	30.7 – 42.3	
- Total dependency	25.4	21.1 – 30.2	
Diabetes mellitus			
- Yes	12.3	11.1 – 13.8	0.001
- No	7.4	7.1 – 7.7	
Heart disease			
- Yes	12.0	10.5 – 13.5	0.001
- No	7.5	7.2 – 7.8	
Stroke			
- Yes	22.4	20.3 – 24.8	0.001
- No	7.1	6.8 – 7.4	
Rheumatoid/joint disease			
- Yes	11.9	11.2 – 12.7	0.001
- No	6.8	6.5 – 7.0	
Hypertension			
- Yes	11.3	10.7 – 12.0	0.001
- No	6.7	6.4 – 7.0	
Injury (1 year)			
- Yes	15.0	13.8 – 16.3	0.001
- No	7.0	6.8 – 7.3	

Bivariate analysis with depression as the dependent variable, dependency as the independent variable and all covariates was carried out. The results show that all covariates were eligible for analysis in the multivariate stage because they were significant with *p* values < 0.25. In multivariate analysis, two covariates were not significant (*p* value > 0.05); therefore, they were excluded from the analysis. The final results are shown in Table 4.

**Table 4:** Odds Ratio for depression in elderly people with functional disorders

	Adj OR depression	95% CI	*p*-value
Functional Status:			
- Dependent	ref		
- Mild dependency	2.064	1.882-2.264	0.090
- Moderate dependency	3.971	3.090-5.102	0.794
- Severe dependency	5.730	4.302-7.632	0.001
- Total dependency	4.147	3.231-5.321	0.001
Female	1.206	1.105-1.316	0.001
Work/Retired	1.287	1.176-1.408	0.001
Education			
- University/college	ref		
- No schooling	2.041	1.461-2.858	0.001
- Did not finish elementary school	2.330	1.678-3.235	0.001
- Elementary school	2.207	1.595-3.056	0.001
- Junior high school	1.790	1.267-2.530	0.001
- Senior high school	1.511	1.071-2.132	0.019
Economic status			
- Quintile 5	ref		
- Quintile 1	1.485	1.282-1.720	0.001
- Quintile 2	1.251	1.066-1.468	0.008
- Quintile 3	1.318	1.114-1.558	0.002
- Quintile 4	1.139	0.966-1.344	0.192
Diabetes mellitus	1.473	1.261-1.720	0.001
Heart disease	1.364	1.151-1.616	0.001
Stroke	1.898	1.608-2.239	0.001
Rheumatoid/joint disease	1.590	1.439-1.758	0.001
Hypertension	1.285	1.143-1.386	0.001
Injury (1 year)	1.941	1.727-2.182	0.001

In the multivariate analysis, it was observed that elderly individuals with severe dependence were more likely to be depressed than those without functional/independent disorders after adjusting for sociodemographic factors and disease history. Additionally, the area of residence and marital status did not have a statistically significant effect on the relationship between functional disorders and depression.

This result was then analyzed for confounding variables, but in fact, none of the variables were not confounding factors.

**Fig. 2:**
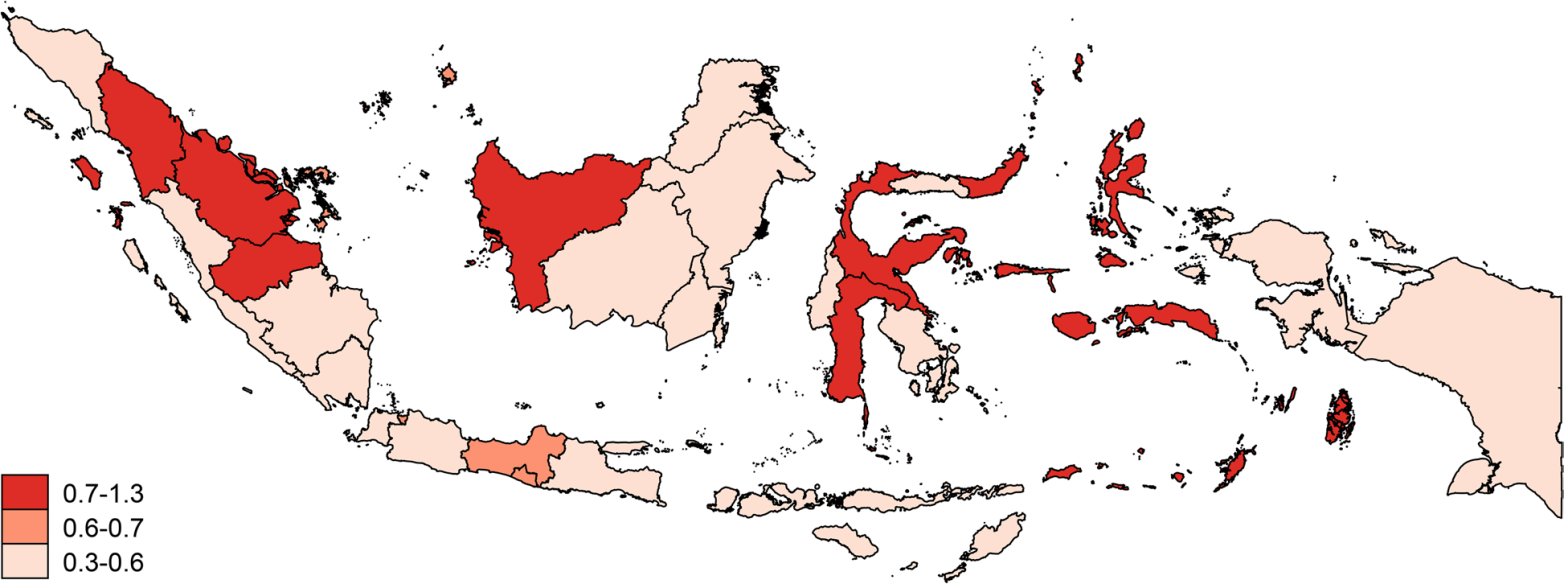
Severe dependency by province

Figure 2 shows the provinces with elderly people with severe dependence on a map of Indonesia consisting of 34 provinces. On the map, several provinces need attention from the local government because they have a high rate of elderly individuals with severe dependence. The provinces in the darkest red color are North Sumatra, Riau, Jambi, West Kalimantan, North Sulawesi, Central Sulawesi, South Sulawesi, North Maluku and Maluku.

## Discussion

According to Table 4, elderly individuals with severe dependence were more likely to be depressed than those without functional/independent disorders. In general, elderly individuals with total dependence have individuals assisting them in their daily activities; therefore, they are able to cope with the condition better. Severe and total dependence is a predictor of psychological problems and quality of life. This is because, in Indonesia, the most important role in determining quality of life is mobility [29]. Therefore, impaired functional status is more of a problem than pain for Indonesians.

Education, socioeconomic level and comorbidities of physical illness affect depression in elderly individuals, but not as much as functional status. Furthermore, sociodemographic factors play a role in certain conditions, for example, falls among the elderly population in Indonesia [30]. Other factors associated with depression among elderly individuals include dementia and marital status [31]. The respondents of this study were elderly individuals living in their homes, generally with their families, but the problem of barriers in the form of poor functional status still needs to be considered, and solutions are needed. One area of importance to elderly individuals in Indonesia is social engagement. This is because they still contribute to the environment around them, for example, by participating in social and religious activities [32]. However, to be able to participate socially in such an environment, sufficient mobility is required, which is an obstacle to those with low functional status. Although the majority of elderly individuals in Indonesia live with their families, with an increasing burden of disease, an idea arises that nursing homes appropriate for the size of Indonesian society are needed. Consequently, several studies have developed an assessment of nursing homes that are expected to suit the wishes of elderly individuals in Indonesia [33, 34].

Although functional status assessments with the Barthel index are widely used in the study of elderly individuals, it is not necessarily appropriate for the individuals to have dementia-related depression [3, 7, 35]. Therefore, it is important to modify the measurement according to the Indonesian context, as in other countries [36–38]. Barthel index validity test in Indonesia have been carried out, but there is not much information regarding this area [28].

In addition, the findings of the study indicate that elderly individuals with physical illnesses have a higher risk of depression than those without such illnesses. Among such diseases, the greatest influence is from stroke and history of injury in the last year, although diabetes, hypertension and joint diseases cannot be ignored. This is because depression occurring comorbid with these physical illnesses will cause a greater burden.

Interventions designed for elderly individuals should implement a multisectoral and comprehensive approach [39]. In Indonesia, an integrated service post for elderly individuals would be suitable. This is because the health program is generally aimed at noncommunicable diseases such as diabetes, stroke, heart disease, chronic obstructive pulmonary disease (COPD) and hypertension, but pain and depression management need to be added [39].

In this study, there was no difference in the proportion of depression between urban and rural areas; however, the place of residency should be distinguished for interventions. For example, in urban areas, social support is needed for elderly individuals, while in rural areas, the most important factor is family support [40]. Furthermore, the results of this study were in line with previous findings, which showed that healthy elderly individuals need social capital, such as good mobility, independence (free from dependence) and good mental health [41].

Further studies are needed on elderly individuals with severe dependence to ascertain the need for other individuals to assist with their daily activities, treat illnesses, and provide social support. Indonesia already has a special integrated service post for elderly individuals that is designed as a vehicle for elderly groups from social, health and other aspects. Furthermore, there is an integrated guidance post (Posbindu) that is more specific to carrying out health checks, especially assessing the risk of noncommunicable diseases. Both posts are easy to access, although the program needs to be improved in terms of both quality and activity [42]. Like other studies, generally suggest the existence of a program of prevention and control of risk factors in the elderly and early detection of depression to prevent a worse impact [43–46].

### Strength and limitation

This is a national study with a large number of respondents covering all areas, including rural and urban areas; therefore, it has good representation. The limitation of this study was cross sectional design that can not explain real relationship of the variables. Another limitation is the fact that this study utilized only data available on from the NHS; therefore, several variables that can affect the output are not taken into account. The variables such family support, social participation, physical activity and other diseases or symptoms for example pain, cognitive impairment, peptic ulcer, migrain, asthma bronchiale .

## Conclusions

The results of this study show that severe functional status has the greatest influence on depression at old age in Indonesia, with the risk being higher for such individuals than for those experiencing total dependence. It was concluded that elderly individuals with impaired functional status experiencing dependence are prone to depression. Furthermore, the physical illness experienced plays a role in the risk of depression, which is higher in individuals with stroke or a history of injury. In addition, sociodemographic factors that play a role include low education and economic levels.

Attention should be provided to elderly individuals with severe dependence since they are not completely disabled but need help to improve their lives physically and psychologically. The government in provinces with a high rate of severe dependence (Fig. 2) should make plans to control this condition. This is necessary because depression will disappear when dependence is minimized and physical illness is treated [4].

## Supplementary Information

**Additional file 1.**

## Data Availability

The datasets generated during and/or analyzed during the current study are not publicly available due to ethical reasons but are available upon request to the Director of the NIHRD.

## Abbreviations

ICD10: International Statistical Classification of Diseases and Related Health Problems 10th revision
NHS: National Health Survey
NIHRD: National Institute of Health Research and Development
MINI: Mini International Neuropsychiatric Interview
OR: Odds ratio
